# Specular Detection on Glossy Surface Using Geometric Characteristics of Specularity in Top-View Images

**DOI:** 10.3390/s21062079

**Published:** 2021-03-16

**Authors:** Seunghyun Kim, Moonsoo Ra, Whoi-Yul Kim

**Affiliations:** 1 Department of Electronics and Computer Engineering, Hanyang University, Seoul 04763, Korea; shkim15ielab@gmail.com; 2 LightVision Inc., Seoul 04793, Korea

**Keywords:** specularity estimation, line detection, gradient vector

## Abstract

In an autonomous driving assistance system (ADAS), top views effectively represent objects around the vehicle on a 2D plane. Top-view images are therefore widely used to detect lines in ADAS applications such as lane-keeping assistance and parking assistance. Because line detection is a crucial step for these applications, the false positive detection of lines can lead to failure of the system. Specular reflections from a glossy surface are often the cause of false positives, and since certain specular patterns resemble actual lines in the top-view image, their presence induces false positive lines. Incorrect positions of the lines or parking stalls can thus be obtained. To alleviate this problem, we propose two methods to estimate specular pixels in the top-view image. The methods use a geometric property of the specular region: the shape of the specular region is stretched long in the direction of the camera as the distance between the camera and the light source becomes distant, resulting in a straight line. This property can be used to distinguish the specular region in images. One estimates the pixel-wise probability of the specularity using gradient vectors obtained from an edge detector and the other estimates specularity using the line equation of each line segment obtained by line detection. To evaluate the performance of the proposed method, we added our methods as a pre-processing step to existing parking stall detection methods and investigated changes in their performance. The proposed methods improved line detection performance by accurately estimating specular components in the top-view images.

## 1. Introduction

**Figure 1:**
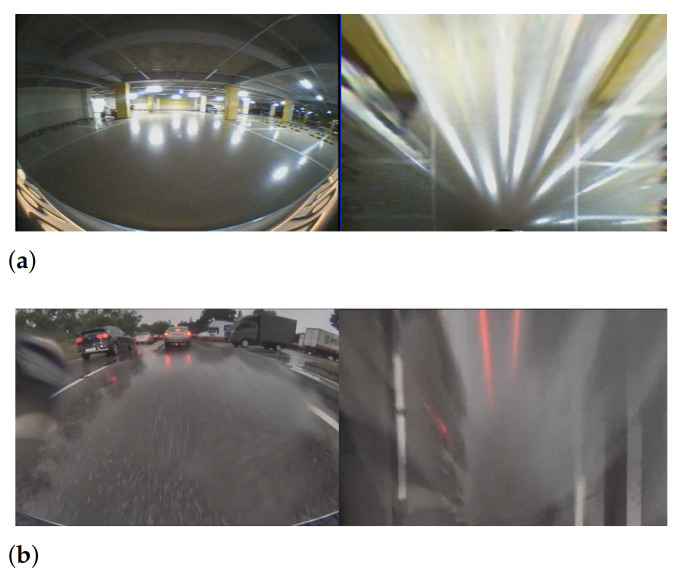
Examples of specularity in top-view images: (**a**) indoor parking lot and (**b**) outdoors on a wet road. The left image is the raw image and the right image is the top-view image.

Perspective geometry of a camera causes planar surfaces such as a parking-lot floor to distort into peculiar shapes. Inverse perspective mapping [1] is a method to correct these irregularities that have been caused by factors such as lens distortion, and imaging geometry by employing an appropriate nonlinear mathematical model. Top-view images, also known as a bird’s-eye view images, generated using the inverse perspective mapping have been widely used in autonomous driving assistance system (ADAS) applications. Because the top-view image provides an elevated view of objects from above, users can easily recognize empty spaces or objects around the vehicle. In conjunction with line detection methods, the top-view images play an important role in line keeping and parking assistance systems. A line response map used for line detection is a filtered image to enhance line elements in the top-view image. To obtain the line response map, two approaches are generally used: edge filter-based [2,3,4,5,6,7,8,9,10] and line filter-based [11,12,13,14,15,16,17]. Both approaches generate the line response map by using the characteristics of a line with a convex intensity profile distinct from the background of the image. The line is then detected from the line response map using a random sample consensus (RANSAC) [8,9,11,12,17,18], Hough transform [3,4,5,6,7,16,19,20], or Radon transform [2,13]. The process described above has been successful in many line detection studies. However, the existing line detection methods produce false detection results when specular reflections are present on the ground, because the specular reflections in the image are similar to that of actual lines. As shown in Figure 1, specular regions generated by light sources form long straight lines. This phenomenon makes line detection very difficult, especially on glossy surfaces on which large bright specular regions form easily. Compounding the problem, some specular patterns produce stronger responses than actual lines in conventional detection methods, because they are much brighter than actual parking lines.

Although researchers have previously tried to remove specular regions from images [21,22,23,24,25,26,27,28], they have focused on distinguishing the specular regions from the background by using intensity or color profiles. Data-driven deep learning-based methods have recently been applied to this problem [29,30], but these methods are not feasible as a pre-processing step, because they require high-end graphic cards to ensure a real-time processing. The deep neural network with 1.9 million parameters takes about 20 ms and 120 ms to produce outputs for 256 × 256 input images with NVIDIA GTX Titan X GPU and Intel i7-6700K CPU, respectively [31]. Since widely used backbones such as VGG 16 or ResNet have more than 20 million parameters, in the line detection applications, the computational cost of the pre-processing might become much higher than that of the main processing. Hence, characteristics of the specular reflections must be investigated to overcome these obstacles.

In general, light sources such as traffic lights, street lights, car headlights, and indoor lighting in parking buildings tend to have oval-shaped specular regions on the floor when they are reflected by an inhomogeneous surface [32]. The distance between a light source and an observer affects the shape of specular regions. As the distance becomes greater, its shape stretches toward the observer such as a stretching shadow. If the distance is sufficiently great, a specular region on the ground resembles a line toward the camera in a top-view image. Morgand et al. [33,34,35] empirically modeled this phenomenon under the name JOint LIght-MAterial Specularity (JOLIMAS), to investigate the way in which a point light source forms a conic shaped specular region, using the Phong model [36].

Two pre-processing methods are proposed to identify false lines due to the reflected light from the light source on the glossy floor by using a geometric property that the specular region appears as a straight line toward the camera in the top-view image. The first generates a probability map that is part of the specularity. Specifically, considering the camera location in the top-view image, the possible direction of specularity can be identified, and at this time, the probability that the pixel is a specular is calculated using gradient vector indicating the direction of intensity changes around the pixel. The second directly applies the geometric property of the specularity to line segments, producing a binary output. False line segments generated by specularity are filtered by investigating a direction of each line segment. Although this method needs processed data as an input, it incurs a low computational burden, and the results of the detection are intuitive. We applied our methods to existing parking stall detection methods as a pre-processing step. Manually collected outdoor and indoor sequences were used to investigate the changes in quantitative performance of the line detection of these methods. There are two versions of each parking stall detection method: one is the original version and the other is an enhanced version, including our methods for suppressing specularity. Our two methods improved the line detection performance in real-world applications. To the best of our knowledge, ours is the first attempt to tackle this issue in ADAS applications. The contributions of this paper are as follows:

1. The geometric property of specularity is used to identify specular pixels. We tried to use the orientational characteristic of the specularity in the image to overcome the limitations of color-based approaches in the line detection.
2. Two methods which can be applied to the line detection using the top-view image are presented. Our two methods estimate the specularity using the intermediate outputs of line filter-based and edge-based line detection approaches.
3. The proposed methods were tested using real-world applications and environments. We applied our methods to existing parking stall detection methods, to demonstrate that our methods can remove artifacts caused by the specularity in various environments.

The rest of the paper is organized as follows. Related studies into removing specular regions from images are summarized in Section 2. Section 3 explains the properties of specular regions and describes the proposed specularity estimation methods. Section 4 presents the experimental results, and the paper is concluded in Section 5.

## 2. Related Works

The existing literature addressing the problem of false detection caused by specularity can be categorized into two approaches: threshold-based [21,22,23,24,25,26,27,28] and deep learning-based [29,30].

### 2.1. Threshold-Based Approaches

These approaches use the intensity or color property of specular regions in images. They are assumed to be outliers with color values distinct from those of other objects in the image.

Saint-Pierre et al. [21] presented a method to remove specular regions in thoracoscopic images. The saturation value of an HSV (hue, saturation, value) channel is used in a pre-processing, to enhance the specular elements in the image. Subsequently, the bump part at the end of the histogram of each RGB (red, green, blue) channel is considered specular.

Tchoulack et al. [22] tried to extract specular regions in endoscopic images via a threshold value obtained from the maximum intensity values in grayscale images and saturation values in the HSV color space.

Chang et al. [23] removed glossy reflections in the portrait photography. This method finds a face region in the photograph using a skin color information. A threshold value is then determined using the maximum and minimum values in the YCbCr color space.

Karapetyan and Sarukhanyan [24] proposed a method to detect specular regions in endoscopic images. Each histogram of local patches obtained from a sliding window is used to determine an adaptive threshold value. For more precise results, this method refines the detected specular region using information about its size and shape.

Morgand and Tamaazousti [25] used the HSV color space to detect specular regions. As a pre-processing step, iterative contrast equalization is performed to make the average intensity value of the image less than a pre-defined intensity value. Next, two threshold values, one calculated from the saturation channel values and the other calculated from the value channel, are applied to identify the specular regions.

Guo et al. [27] presented a method to suppress specular regions in endoscopic images. The method uses grayscale images and creates binary images using a predefined threshold to extract the specular regions. A dilation technique is then used to refine the extracted regions.

Silva et al. [28] performed contrast enhancement using the maximum and minimum values for grayscale images as a pre-processing step for detecting specular regions. The specular regions are extracted using a threshold value obtained from the statistical information of the image.

Li et al. [26] used two reference images, sparse and highlight, to provide specularity information. The sparse image obtained using robust principal component analysis (RPCA) contains a rough estimation of the specular region and the highlight image indicates the predicted specular region using two threshold values. If a similarity between the sparse and highlight image exceeds a certain threshold, the raw image is reconstructed from low-rank image acquired using RPCA. If not, iterative optimization using adaptive RPCA is applied to modify low-rank and sparse images.

### 2.2. Deep Learning-Based Approaches

These approaches have been reported more recently than the threshold-based approaches. Various types of a network have been designed for different applications. Backbones of the network have been shown to generate more powerful features to distinguish objects than traditional hand-crafted features.

Rodriguez-Sanchez et al. [29] used two segmentation networks to extract specular regions in the image. The first network extracts specular regions, and second network reconstructs the extracted specular regions.

Funke et al. [30] proposed a SpecGAN based on a generative adversarial network (GAN) specialized for removing specular regions from images. The SpecGAN adds self-regularization loss to make the generator only change the specularity region. Some of the terms of cycle consistency loss have been adopted to achieve a similar effect to the paired training.

The deep learning-based methods are state-of-the-art approaches that are widely used for computer vision applications such as object detection, classification, and semantic segmentation. They could be applied to classify specular regions based on trained features of convolutional neural networks. However, aside from the computational issue, the deep learning-based methods have a critical drawback that they can produce unexpected results for the input with different characteristics from the training dataset [37,38]. The threshold-based approaches are suitable for the pre-processing, due to their low computational cost. However, it is impossible to distinguish between specular regions and objects with similar color properties. If the specular regions reflected on the glossy floor have the same brightness or color as the surrounding objects in the parking lot or clearly distinguished parking lines, it is difficult to solve with the existing methods based on the thresholding. To address this problem, we propose new methods to distinguish the specular regions using the geometric property of the specularity. The proposed methods are highly effective for detecting lines in images with specular regions.

## 3. Proposed Method

Our proposed methods use a geometric clue; the specular regions are stretched toward the camera. Although this geometric clue can be observed in any scene captured by a camera, we set our target domain as top-view images. We chose this domain because the location of the camera in the top-view image can be obtained in generating the top-view image, and the specular regions are overstated in top-view images and can significantly hinder the line detection. In this section, the geometric property of specularity is explained, and the way in which we implement this concept to estimate specularity is discussed in detail.

### 3.1. The Geometric Property of Specularity

**Figure 2:**
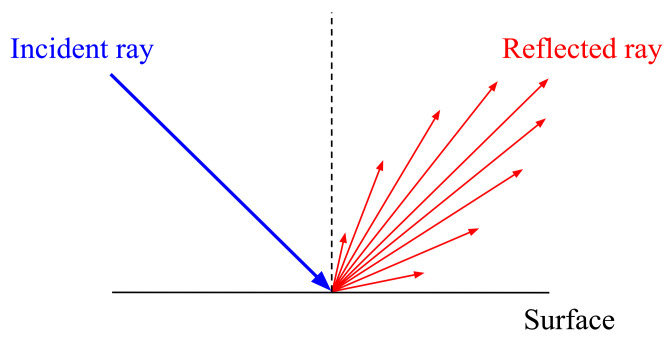
An illustration of reflection from a glossy surface.

From a macroscopic point of view, there are two terms used to describe reflection: specular or diffuse. Specular reflection means that the light rays reflect at the same angle as the angle of incidence, whereas diffuse reflection means that the light rays scatter equally in every direction. In real-world situations, both types are ideal and rarely happen in isolation. Reflections from most existing materials have mixed characteristics, being neither completely specular nor completely diffuse. Phong [36] empirically modeled reflections using specular and diffuse terms. According to the model, a reflection from a glossy surface such as a wet road or a floor coated with the epoxy or concrete, which is slightly rough at a microscopic level, is very close to the specular reflection but has a small amount of scattering (Figure 2).

**Figure 3:**
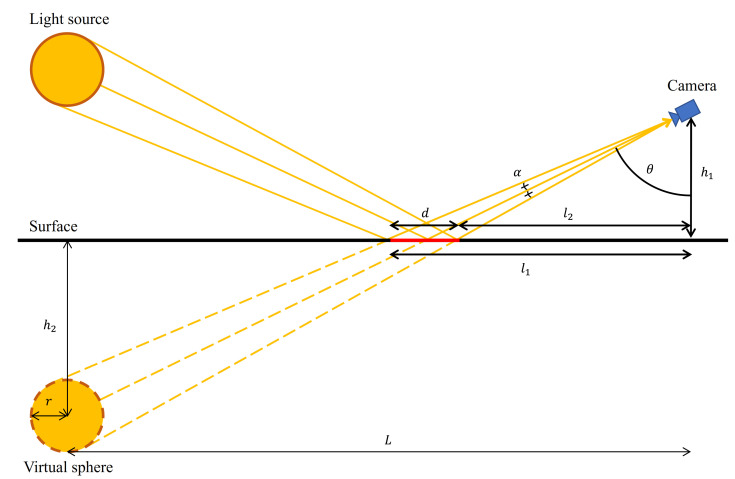
Length of the major axis according to the position of the light source and camera.

Since specularity caused by this reflection produces a striking effect in the image, many researchers have tried to detect and remove this effect, as mentioned in Section 2. However, there is a limitation to what can be achieved. Using only the color properties of the reflection, objects with a color similar to that of the specular region cannot be distinguished. To solve this problem, we handle specularity from a geometric point of view, considering the JOLIMAS model, in which the shape of the specularity based on Phong’s model is analyzed. In the JOLIMAS model, the shape of the specular region is an orthogonal projection of a virtual sphere located at a point symmetrical to the light source relative to the ground (Figure 3), in which a conic section generally forms. The major axis of the specular region can be calculated as

(1) $$\begin{array}{cc} d=l_{1}-l_{2} & =h_{1}\left\{\tan\left(\theta +\alpha \right)-\tan\left(\theta -\alpha \right)\right\}=\frac{2h_{1}r\sqrt{L^{2}+{\left(h_{1}+h_{2}\right)}^{2}-r^{2}}}{{\left(h_{1}+h_{2}\right)}^{2}-r^{2}}, \end{array}$$

where

*d* is the major axis of an ellipse from the projection of the virtual sphere,
θ is the reflection angle of the ray emitted from the center of the light source,
*L* is the horizontal distance between the virtual sphere and the camera,
*r* is the radius of the virtual sphere,
$l_{1}$ is the horizontal distance between the camera and the vertex farthest away from the camera,
$l_{2}$ is the horizontal distance between the camera and the vertex closest to the camera,
$h_{1}$ is the height between the camera and the surface,
$h_{2}$ is the height between the virtual sphere and the surface,
*a* is angle between two lines; one is a tangent line to the virtual sphere from the camera and the other is a line passing through the center of the virtual sphere from the camera.

Since *d* increases as *L* increases, the specular region stretches toward the camera direction in the image, while retaining its width. A length of *d* in the image changes according to a projection angle between the image plane and camera plane. This length is proportional to cosine θ and maximized when θ is zero; this represents the top-view image. Because the inverse perspective mapping removes a perspective effect caused by different depths of objects [39], the specular region which looks like a slightly stretched ellipse in the oblique-view image appears as a very long straight line in the top-view image.

### 3.2. Pixel-Wise Specularity Estimation

**Figure 4:**
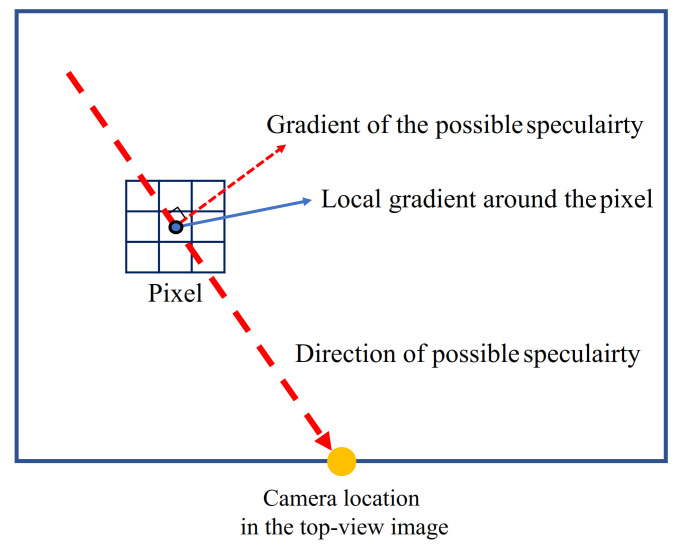
Example of a local gradient around the pixel and gradient of the possible specularity.

Because the intensity values and profiles of the lines and specular regions are very similar, the latter can induce false line detection results. The specularity inducing the false line detection has a linear shape due to a long distance between the camera and light source. According to the geometric property discussed in Section 3.1, its orientation is directed toward a camera location in the top-view image. Since the camera location in the top-view image can be obtained during the extrinsic calibration process to define a geometric relationship between the camera and ground plane, we do not need an additional step to acquire the camera location in the top-view image. Therefore, we can predict all possible stretched directions of the specularity at a certain pixel. This is an important clue to distinguish specular pixels and non-specular pixels. If a pixel is specular, a local gradient around the pixel should be similar to the gradient of the possible specularity. These two gradients can be obtained using intensity changes of adjacent pixels and direction of the possible specularity as depicted in Figure 4.

**Figure 5:**
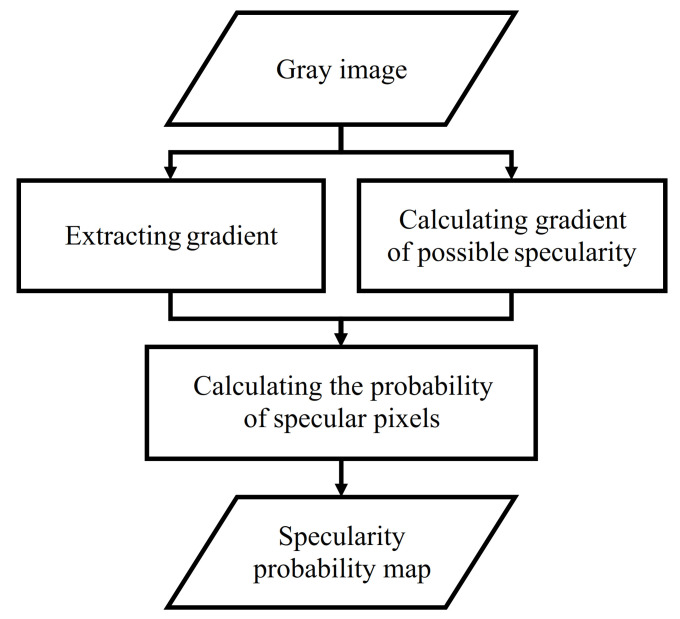
A flowchart of the probability map generation to estimate specular pixels.

Due to the quantization error caused by the process of generating top-view images and the non-spherical shape of the light source, it is difficult to determine specular pixels discretely. Therefore, a probability map indicating pixel-wise probability that a pixel is specular is generated to fill this gap. The information from the probability map is then used to suppress specular pixels. There can be many possible applications using this map to suppress specular pixels. As an example, we describe how to apply this probability map to other applications in Section 4. A flowchart of the way in which the probability map is generated is depicted in Figure 5.

#### 3.2.1. Extracting Gradient

To obtain a local gradient around a pixel, vertical and horizontal gradient maps are first extracted by applying a 3 × 3 Sobel filter to a grayscale top-view image. This process can be expressed using the following equations:

(2) $$\begin{array}{c} E_{h}=G⊛S_{h},\;E_{v}=G⊛S_{v}, \end{array}$$

where $E_{h}$ is the horizontal gradient map, $E_{v}$ is the vertical gradient map, *G* is the grayscale top-view image, $S_{h}$ is a Sobel horizontal edge detector, and $S_{v}$ is a Sobel vertical edge detector. Then the gradient vector $N_{I}\left(x\right)$ at pixel x=(u,v) is calculated as

(3) $$\begin{array}{c} N_{I}\left(x\right)=(\frac{E_{h}\left(x\right)}{\sqrt{E_{h}{\left(x\right)}^{2}+E_{v}{\left(x\right)}^{2}}},\frac{E_{v}\left(x\right)}{\sqrt{E_{h}{\left(x\right)}^{2}+E_{v}{\left(x\right)}^{2}}}). \end{array}$$

$N_{I}\left(x\right)$ is a 2D vector indicating the direction of the local gradient around the pixel x. The $N_{I}\left(x\right)$ is used to distinguish specular pixels in subsequent work.

#### 3.2.2. Calculating Gradient of Possible Specularity

**Figure 6:**
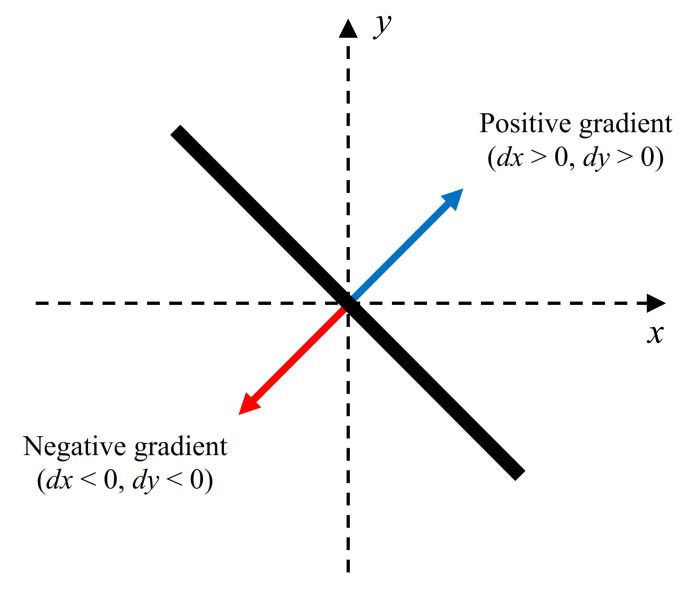
Two types of gradient for a line segment.

Specularity appears as radial lines centered on the camera position in the top-view image. This geometric property gives us a clue with which to distinguish between specular and non-specular pixels. The gradient vector of the possible specularity at a certain pixel can be calculated using the camera location. However, as shown in Figure 6, a straight line can have one of two gradient vectors: positive or negative. Therefore, one specular line can have either positive gradient or negative gradient vector according to the direction of intensity changes. To take this into account, we calculate the two gradient vectors, $N_{R}^{+}\left(x\right)$ and $N_{R}^{-}\left(x\right)$, from the possible specularity direction at pixel x. These two vectors are defined by following equations:

(4) $$\begin{array}{c} N_{R}^{+}\left(x\right)=(\frac{v}{\sqrt{{\left(u-u_{c}\right)}^{2}+{\left(v-v_{c}\right)}^{2}}},\frac{u}{\sqrt{{\left(u-u_{c}\right)}^{2}+{\left(v-v_{c}\right)}^{2}}}),\\ N_{R}^{-}\left(x\right)=(\frac{-v}{\sqrt{{\left(u-u_{c}\right)}^{2}+{\left(v-v_{c}\right)}^{2}}},\frac{-u}{\sqrt{{\left(u-u_{c}\right)}^{2}+{\left(v-v_{c}\right)}^{2}}}), \end{array}$$

where $\left(u_{c},v_{c}\right)$ is the pixel location of the camera in the top-view image.

#### 3.2.3. Calculating the Probability of Specular Pixels

A probability map P(x) represents the probability of a specular pixel at location x. A 2D Gaussian function is used to calculate the probability, as follows:

(5) $$\begin{array}{c} G_{\sigma }(∥x-m∥)=\exp\left(-\left(\frac{{\left(u-m_{u}\right)}^{2}}{2\sigma_{u}^{2}}+\frac{{\left(v-m_{v}\right)}^{2}}{2\sigma_{v}^{2}}\right)\right), \end{array}$$

where $m=(m_{u},m_{v})$ is the mean and $\sigma =(\sigma_{u},\sigma_{v})$ is the standard deviation of x. To generate the probability map, we consider not only the gradient vectors obtained, but also the local spatial continuity, because the specular pixels form a straight line of a specific width. The concept of a bilateral filter [40] was adopted to combine the spatial and gradient information. Consequently, the equations calculating the values of the positive and negative probability maps, $P^{+}\left(x\right)$ and $P^{-}\left(x\right)$, are defined as follows:

(6) $$\begin{array}{c} P^{+}\left(x\right)=\frac{1}{W_{s}}\sum_{x_{i}\in S}G_{\sigma_{s}}(∥x_{i}-x∥)G_{\sigma_{n}}(∥N_{I}\left(x_{i}\right)-N_{R}^{+}\left(x_{i}\right)∥), \end{array}$$

(7) $$\begin{array}{c} P^{-}\left(x\right)=\frac{1}{W_{s}}\sum_{x_{i}\in S}G_{\sigma_{s}}(∥x_{i}-x∥)G_{\sigma_{n}}(∥N_{I}\left(x_{i}\right)-N_{R}^{-}\left(x_{i}\right)∥), \end{array}$$

(8) $$W_{s}=\sum_{x_{i}\in S}G_{\sigma_{s}}(∥x_{i}-x∥),$$

(9) $$\begin{array}{c} S=\{x|x:\left(u,v\right),\left(u_{o}-w\right)\leq u\leq \left(u_{o}+w\right),\left(v_{o}-w\right)\leq v\leq \left(v_{o}+w\right)\}, \end{array}$$

where $\left(u_{o},v_{o}\right)$ is the center of a predefined window, *w* indicates half of the predefined window size, $W_{s}$ is a normalization term which limits the range of the probability values from 0 to 1, and $\sigma_{s}=\left(\sigma_{u}^{s},\sigma_{v}^{s}\right)$ and $\sigma_{n}=\left(\sigma_{u}^{n},\sigma_{v}^{n}\right)$ are constant vectors selected manually to adjust the size of each Gaussian kernel. According to the type of an application, the two probability maps can be used separately or in combination.

**Figure 7:**
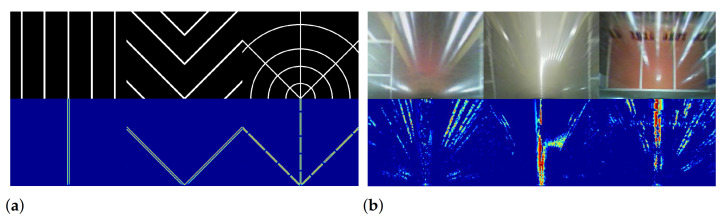
An example of applying probability maps: (**a**) synthetic images and (**b**) real images. The first row shows the raw images and the second row shows pseudo colored probability maps.

We generated probability maps using synthetic and real images for qualitative evaluation. The set operation ‘or’ was used to combine the positive and negative probability maps. The combined probability maps are depicted in Figure 7; the red pixels indicate higher probabilities of being specular pixels than blue pixels. The probability map helped to identify the specular pixels correctly.

**Figure 8:**
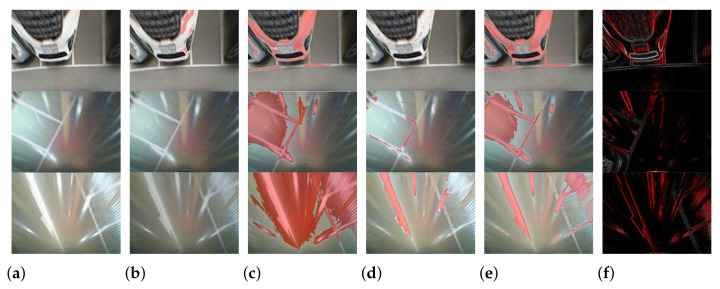
Results of qualitative evaluation for specularity detection: (**a**) raw image, (**b**) Morgand et al. [25], (**c**) Chang et al. [23]. (**d**) Li et al. [26], (**e**) Silva et al. [28], and (**f**) proposed.

We qualitatively compared the proposed probability map with the results of other existing specularity detection methods (Figure 8). The methods published by Morgand et al. [25], Chang et al. [23], Li et al. [26], and Silva et al. [28] were used for the comparison; in Li’s method [26], we used the result of specular highlight detection (highlight images) for the comparison. These methods assume that specularity has an intensity value distinct from that of other objects in the image and determine threshold values using statistical information about pixel intensity values. Thresholding is applied to extract specular regions from the image. Although the main target of the alternate methods is slightly different from that of our work, which detects the edge of the specularity, this comparison makes it clear that the color-based approaches cannot be directly applied to the line detection. Since the color-based methods have a lower power to discriminate between the specularity and white bumper or parking lines, they remove details which must be preserved for the line detection. However, the proposed probability map correctly estimates most specularity edges, while preserving the edges of the white parking lines. This comparison indicates that the geometric property of specularity is effective in distinguishing specular from non-specular pixels. Because this result shows the superiority of our method for line detection, we did not perform additional comparisons using color-based approaches.

### 3.3. Line-Segment-Level Specularity Estimation

In the previous section, we described the process of generating the probability map to estimate specular pixels. To apply this map to other applications, gradient information is required to calculate the probability of specularity of each pixel. Since the line filter-based methods do not use edge detector to extract line responses, the pixel-wise specularity estimation cannot be directly applied to these methods. To address this issue, we developed a line-segment-level specularity estimation using line segments, which is the intermediate output of the line filter-based methods. For this estimation, the line equation of each line segment is used to determine specularity. If a line passes through the center location of the camera, the corresponding line segment is regarded as specular. To consider the quantization error described in the previous section, we give an extra margin to a tolerance range used for this estimation. Specularity can be determined as follows:

(10) $$S\left(i\right)=\left\{\begin{array}{cc} 1, & \mathrm{if}\;v_{c}-M\leq l_{i}\left(u_{c}\right)\leq v_{c}+M\\ 0, & \mathrm{otherwise} \end{array}\right.,$$

where *S* indicates specularity, *i* is the index of line segments, $l_{i}$ is the line equation of the *i*th line segment, $\left(u_{c},v_{c}\right)$ is the camera location in the top-view image, and *M* is a pre-defined margin.

## 4. Experiments

**Figure 9:**
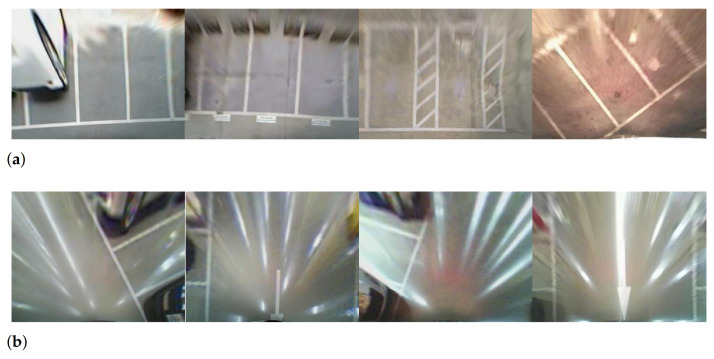
Example images: (**a**) outdoor and (**b**) indoor.

To evaluate the performance of the proposed methods, we applied them to existing parking stall detection methods [8,15] as a pre-processing stage. Because there is no publicly available database for evaluating parking stall detection performance, a private database was constructed from images captured in parking lots with various patterns and environments. This database consists of indoor and outdoor parking lot sequences with rectangular parking stalls and was captured with a fisheye camera with 720 × 480 resolution and 30 fps mounted on the rear bumper of a vehicle. To avoid data duplication, each sequence was subsampled once every 15 frames. The parking scenario and parking place were changed to obtain various situations and specular patterns as shown in Figure 9. We collected 12 sequences: six sequences were taken outdoors and the rest were captured indoors. The viewpoint of the database was changed to top-view at 400 × 300 resolution; one pixel in the top-view image corresponds to 2 cm in the real world. The dataset specifications are summarized in Table 1.

### 4.1. Performance Evaluation Metrics

**Figure 10:**
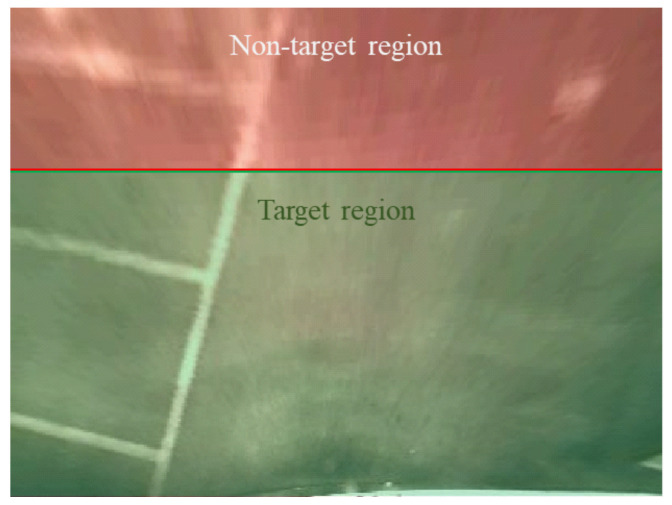
Example image for quantitative evaluation. The target region is green, and the non-target region is red.

The two proposed methods focus on detecting elements caused by specularity in the intermediate output generated during the line detection process. The pixel-wise probability map provides probabilistic information for the edge of a specular region, and the line-segment-level estimation identifies specular line segments among detected line segments generated by the line detection. Since the goal of these two methods is not to estimate all specular regions, the performance of existing parking stall detection methods was measured by comparing junction detection results when the proposed specularity suppression methods were applied at the pre-processing stage, and when it was not. Let us define the junction as an intersection between a horizontal and vertical parking lines. Assume that we have detected a junction by using a parking stall detection method. Junctions located within a 10-pixel radius from the ground truth are considered to be positive, and all others are negative. Because the quality of top-view images degrades as the distance increases from the camera, we only consider junctions close to the camera in the performance evaluation. Junctions whose distance from the camera is less than 200 pixels in the y-axis are our main target (Figure 10). The well-known definitions of precision and recall are used as performance metrics. These measures are calculated as:

(11) $$\begin{array}{c} Precision=\frac{\#\mathrm{of}\mathrm{ground}\mathrm{truth}\mathrm{in}\mathrm{positive}\mathrm{samples}}{\#\mathrm{of}\mathrm{positive}\mathrm{samples}},\\ Recall=\frac{\#\mathrm{of}\mathrm{ground}\mathrm{truth}\mathrm{in}\mathrm{positive}\mathrm{samples}}{\#\mathrm{of}\mathrm{ground}\mathrm{truth}\mathrm{samples}}. \end{array}$$

### 4.2. Quantitative Analysis

#### 4.2.1. Implementations

We reproduced two existing parking stall detection methods: Lee’s method [15] and Suhr’s method [8]. These methods were implemented as described in the original paper, with missing values filled with values chosen to produce similar results to the original paper.

**Figure 11:**
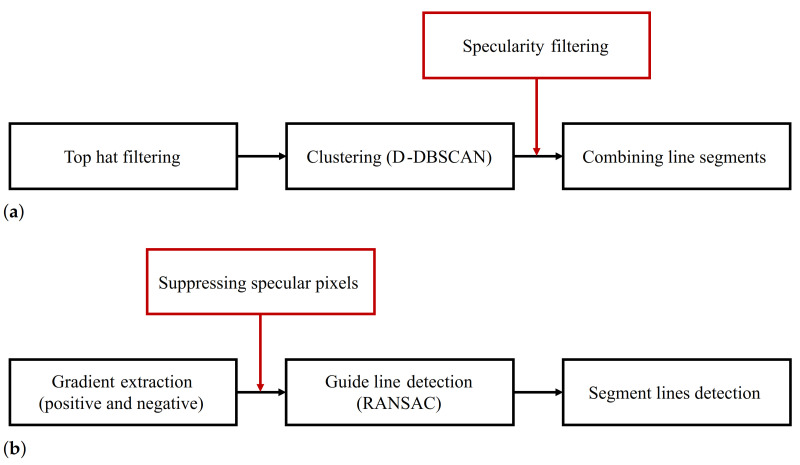
Flowcharts of (**a**) Lee’s method [15] and (**b**) Suhr’s method [8] with added specularity suppression and filtering. The black box is the original process, and the red box indicates the addition of our method.

Lee’s method adopts the line-filter-based approach to detect parking lines in the top-view image. It generates a line response map from the grayscale image using a top-hat filter. Since this filter is symmetrical and designed to extract center points of a parking line with a certain thickness, gradient information is not obtained during this process. It is, therefore, redundant to apply an additional edge filter to generate the specularity probability map. In this case, the line-segment-level estimation can be used to filter out specularity. We added this process between the clustering and line segment combining processes, as depicted in Figure 11a. Line segments acquired from the clustering process are inspected to filter the specularity. Then, the filtered line segments considered to be non-specular are passed to the next process. The filtering process incurs a low computational cost because it uses information already obtained—the line equation and line segments—from the clustering process; all that remains is to solve a linear equation to determine specularity.

Suhr’s method is an edge-based parking line detection method using positive and negative gradients. Since this method obtains gradient information to extract the line response, the probability map can be generated from this intermediate output. Specularity suppression using the probability map is applied to the process of guide line detection. Grayscale images and Sobel filters are used for the extraction of positive and negative gradients. RANSAC is then applied to each the positive and negative gradient map to detect guide lines, with the specularity suppression process placed between the gradient extraction and RANSAC processes, as depicted in Figure 11b. At the voting stage in RANSAC, different weight values are allocated, according to the probability of pixels being specular.

#### 4.2.2. Quantitative Results

Table 2 summarizes the quantitative results using Lee’s method [15] for 12 sequences with different specularity patterns and environments. In Table 2, ‘enhanced’ means that our specularity estimation was applied and ‘baseline’ means that it was not applied. These terms are also used in Table 3 later.

**Figure 12:**
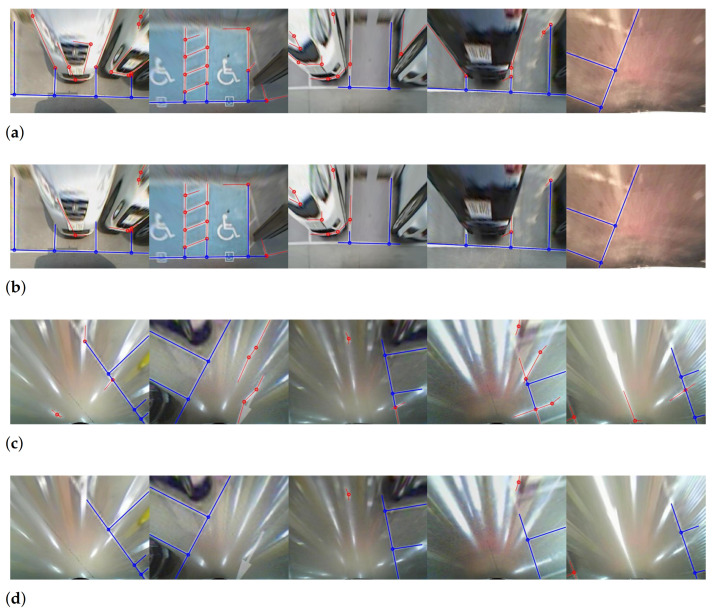
Comparison of junction detection results using Lee’s method [15]: (**a**) outdoor (baseline), (**b**) outdoor (enhanced), (**c**) indoor (baseline), and (**d**) indoor (enhanced). Solid blue lines are true positives and solid red lines are false positives. Dots indicate the junction of two connected line segments.

The enhanced version has precision values improved by up to 29.8% in most sequences. This result indicates that our method successfully detects specularity and reduces false positives. Since the indoor sequences contain specularity, the enhanced version shows better performance in these images. However, the proposed approach also shows higher precision when applied to the outdoor sequences. If the specularity filtering worked as intended, there should be no difference in precision and recall for the outdoor sequences, which do not contain specularity. A discrepancy arises because of the distortion caused by objects standing perpendicular to the ground in the top-view image. Although in theory the background is different, the edges of these objects are also oriented towards the camera location, in the same manner as specularity [8]. Therefore, line segments generated from these objects are filtered out by the specularity filtering. Examples of this case are presented in first and fourth columns in Figure 12a,b.

Figure 12 shows the detection results for outdoor and indoor sequences. The false positives generated by car bumpers or specularity are removed by the proposed specularity filtering. The filtering does not affect most true positives; the parking lines detected by the baseline approach are also detected in the enhanced version. This finding implies that our line-segment-level specularity estimation can be successfully applied to line filter-based approaches without significant additional computational cost.

There was no improvement for recall, because Lee’s method finds all line segments in the top-view image. Lee’s method combines the line segments by considering geometric constraints. Then, the structural information containing a distance between adjacent junctions and each direction of line segments from a connected junction is used to detect parking stalls. In this algorithm, specularity filtering cannot help in finding missing true positives in line detection. However, it may be important for parking stall detection. Comparing the fifth column in Figure 12c,d, the enhanced version provides more distinguishable structural information about actual parking stalls; the approach implicitly assists in the estimation of parking stall structure.

**Figure 13:**
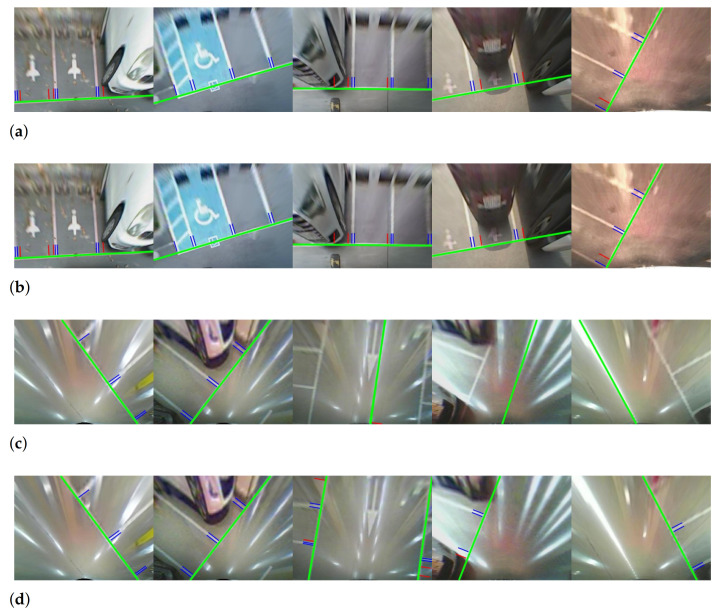
Comparison of junction detection results using Suhr’s method [8]: (**a**) outdoor (baseline), (**b**) outdoor (enhanced), (**c**) indoor (baseline), and (**d**) indoor (enhanced). Solid green lines are detected guide lines. Solid blue lines are true positives and solid red lines are false positives.

To test the effectiveness of the proposed pixel-wise specularity estimation, we applied it to Suhr’s method [8] for guide line detection, to suppress specular pixels. The result of quantitative evaluation is presented in Table 3. It shows that the Suhr’s method becomes robust to the specularity by using our probability map to estimate specular pixels. In all indoor sequences, recall values were the same as or better than the baseline. The recall increased significantly, from 35.98% to 73.78% for 9 sequences, and from 14.75% to 69.25% for 12 sequences. Two versions of Suhr’s method produced very similar recall and precision in outdoor sequences with no specularity (Figure 13a,b). The results using outdoor and indoor sequences implies that the specularity suppressing process using the proposed probability map has very little effect on non-specular pixels and suppresses most of the specular pixels in the top-view image.

However, the recall of the enhanced version is almost same as that of the baseline in some indoor sequences. This phenomenon occurs because Suhr’s method locates parking stalls by sequentially detecting two types of lines: guide lines and segment lines. Suhr’s method first detects a guide line and then finds segment lines based on the detected guide line information. To detect the guide line, his method uses RANSAC and considers a line with the maximum inlier ratio to be a guide line. This scheme shows different performance according to the pattern of specularity. If specular pixels are widely scattered and form many short lines, the line response of the actual parking line is dominant over specularity. In this case, the baseline correctly detects the guide line, and recall is the same in both versions, as shown in first and second column of Figure 13c,d. The baseline has a very weak guide line detection performance for a specularity pattern in which specular pixels are densely gathered to form a strong straight line. The third to fifth columns of Figure 13c,d show the cases where the baseline fails to detect a correct guide line due to the strong specularity. The enhanced version therefore shows noticeably improved recall only in the 9 and 12 sequences captured from the environment in which long straight specularity is present.

**Figure 14:**
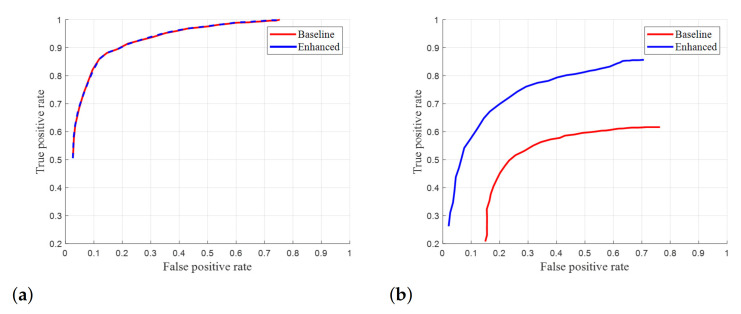
ROC curve comparison between the ’baseline’ and ’enhanced’ Suhr’s method [8] without and with the proposed method: (**a**) outdoor sequences and (**b**) indoor sequences.

ROC curves were calculated by changing the threshold for adjusting the sensitivity of segment line detection. Figure 14a,b are ROC curves for outdoor and indoor sequences, respectively. The enhanced version shows similar performance to the baseline in outdoor sequences and better performance in indoor sequences. The same tendency is seen in Table 3, indicating that our method successfully estimates specular pixels in the top-view image.

#### 4.2.3. Running Time

We measured the running time for generating the probability map and for line-segment-level estimation. All source code is written in C/C++ and runs on a Ryzen7 2700X CPU with 64 GB memory. Generating the probability map takes about 13 ms for processing the 400 × 300 top-view image. This process can take less time if we take advantage of a multi-core environment. In the case of line-segment-level estimation, it requires too little computation to measure processing times. Since our methods run far beyond the real time, these methods can easily be adopted as a pre-processing step for various ADAS applications that use top-view images.

## 5. Conclusions

We proposed specularity estimation methods that can be used as a pre-processing step in line detection applications using top-view images. The geometric property of specularity was used to overcome the limitation of the color-based approach, with which white lines and specular regions cannot be distinguished. The pixel-wise specularity estimation can be applied to edge-based approaches, and the line-segment-level specularity estimation can be applied to line-filter-based approaches. For quantitative evaluation, we used 12 manually collected sequences and two existing parking stall detection methods. The quantitative results showed that our methods successfully estimate specularity and improve the line detection performance in the environment with specularity, while maintaining performance in the environment without specularity. Despite the two existing methods adopt different approach to detect parking stalls, the proposed methods can detect the specularity using the intermediate output of each these approaches. All of the processes to generate the probability map are completed within 13 ms for 400 × 300 image, and the line-segment-level estimation has a much lower computational cost. Our specularity estimation methods can therefore be widely applied to existing line detection methods. In future studies, we intend to set a hyperparameter such as standard deviation, to generate the probability map adaptively, using a mathematical approach, to make our methods more generic.

## Figures and Tables

**Table 1:** Dataset characteristics.

Sequence Number	# of Images	Environment	Specularity
1	56	Outdoor	Absent
2	58	Outdoor	Absent
3	48	Outdoor	Absent
4	60	Outdoor	Absent
5	37	Outdoor	Absent
6	44	Outdoor	Absent
7	42	Indoor	Present
8	99	Indoor	Present
9	52	Indoor	Present
10	44	Indoor	Present
11	41	Indoor	Present
12	87	Indoor	Present

**Table 2:** Quantitative results using Lee’s method [15].

Sequence Number	Precision (Baseline)	Precision (Enhanced)	Recall (Baseline)	Recall (Enhanced)
1	49.43%	57.46%	85.53%	86.18%
2	49.37%	56.52%	82.98%	82.98%
3	70.94%	79.12%	85.21%	85.21%
4	54.81%	57.81%	82.22%	82.22%
5	40.28%	50.60%	75.22%	75.22%
6	100.00%	100.00%	93.33%	93.33%
7	60.75%	83.33%	76.47%	76.47%
8	53.16%	70.79%	69.23%	69.23%
9	57.29%	72.26%	67.07%	68.29%
10	55.80%	65.25%	85.56%	85.56%
11	55.36%	71.59%	72.09%	73.26%
12	50.20%	80.00%	62.12%	62.63%

**Table 3:** Quantitative results using Suhr’s method [8].

Sequence Number	Precision (Baseline)	Precision (Enhanced)	Recall (Baseline)	Recall (Enhanced)
1	53.24%	53.86%	98.94%	98.94%
2	67.49%	67.32%	93.20%	93.20%
3	70.11%	69.92%	98.47%	98.47%
4	33.49%	33.53%	98.60%	98.60%
5	38.29%	37.17%	96.59%	97.16%
6	53.82%	53.82%	97.78%	97.78%
7	83.52%	83.33%	89.41%	91.18%
8	38.13%	37.75%	75.00%	78.30%
9	69.01%	67.79%	35.98%	73.78%
10	83.33%	83.78%	86.11%	86.11%
11	77.85%	76.51%	67.44%	73.84%
12	56.73%	77.81%	14.75%	69.25%
